# Ferroelectric 2D SnS_2_ Analog Synaptic FET

**DOI:** 10.1002/advs.202308588

**Published:** 2024-02-20

**Authors:** Chong‐Myeong Song, Dongha Kim, Shinbuhm Lee, Hyuk‐Jun Kwon

**Affiliations:** ^1^ Department of Electrical Engineering and Computer Science Daegu Gyeongbuk Institute of Science and Technology (DGIST) Daegu 42988 South Korea; ^2^ Department of Physics and Chemistry DGIST Daegu 42988 South Korea

**Keywords:** ferroelectrics, field‐effect transistor, synaptic device, tin disulfide (SnS_2_)

## Abstract

In this study, the development and characterization of 2D ferroelectric field‐effect transistor (2D FeFET) devices are presented, utilizing nanoscale ferroelectric HfZrO_2_ (HZO) and 2D semiconductors. The fabricated device demonstrated multi‐level data storage capabilities. It successfully emulated essential biological characteristics, including excitatory/inhibitory postsynaptic currents (EPSC/IPSC), Pair‐Pulse Facilitation (PPF), and Spike‐Timing Dependent Plasticity (STDP). Extensive endurance tests ensured robust stability (10^7^ switching cycles, 10^5^ s (extrapolated to 10 years)), excellent linearity, and high *G*_max_/*G*_min_ ratio (>10^5^), all of which are essential for realizing multi‐level data states (>7‐bit operation). Beyond mimicking synaptic functionalities, the device achieved a pattern recognition accuracy of ≈94% on the Modified National Institute of Standards and Technology (MNIST) handwritten dataset when incorporated into a neural network, demonstrating its potential as an effective component in neuromorphic systems. The successful implementation of the 2D FeFET device paves the way for the development of high‐efficiency, ultralow‐power neuromorphic hardware which is in sub‐femtojoule (48 aJ/spike) and fast response (1 µs), which is 10^4^ folds faster than human synapse (≈10 ms). The results of the research underline the potential of nanoscale ferroelectric and 2D materials in building the next generation of artificial intelligence technologies.

## Graphical abstract

The ferroelectric HZO‐based SnS_2_ analog synaptic FET exhibits polarization changes in response to electrical signals, altering its electrical characteristics. The resulting electrical characteristic changes of the transistor mimic neuronal behavior. Depending on the direction of polarization, the conductivity of the SnS_2_ channel changes, which is analogous to the inhibition or excitation of signal transmission by neurotransmitters in synapses.

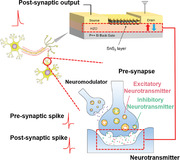

## 1 Introduction

The demand for artificial intelligence (AI) technology and computing power in machine learning has been increasing in parallel with the explosive growth of data‐centric applications.^[^ ^1^ ^]^ In the widely used von Neumann architecture today, the separation of memory and logic areas leads to a bottleneck where data cannot be processed and read simultaneously. The current segregated structure in AI technology used for machine learning and deep learning results in significant waiting time and energy consumption costs due to the need for round trips of large amounts of data between processing and memory devices.^[^ ^2^ ^]^ Data‐centric computing devices require high performance, energy efficiency, and logic‐memory integration capabilities to support various applications. This has led to the emergence of neuromorphic devices that replicate the structure of the human brain, enabling in‐memory computations and providing high energy efficiency and densely merged logic‐memory functionalities through parallel computations and adaptive learning.^[^ ^3^ ^]^ Various devices have been suggested as candidates for implementing neuromorphic computing hardware.^[^ ^4^ ^]^ However, most of them face difficulties in practical applications due to low data retention and nonlinear weight properties. To achieve high accuracy in neuromorphic systems, the development of devices with ideal synaptic characteristics is necessary.^[^ ^5^ ^]^ There are various candidates, such as floating‐gate, trap‐charging, and ferroelectric materials, but stability and variability issues, such as linear/symmetric weight updates and state numbers, remain major obstacles for energy‐efficient neuromorphic computing in practical applications.^[^ ^6^ ^]^ Generally, higher nonlinearity leads to complex weight modulation and incurs high energy and time costs during training. In contrast, linear and symmetric weight update operations with a sufficient number of states can effectively enhance the inference accuracy and reliability of neuromorphic computing.^[^ ^7^ ^]^ Therefore, improving linearity and symmetry in artificial synaptic devices is necessary to construct low‐power, high‐precision artificial neuromorphic networks. Ferroelectrics are expected to solve these problems by solving the issues of linearity and symmetry.^[^ ^8^ ^]^ Ferroelectric materials possess a spontaneous polarization state that is maintained even without an external electric field, and this polarization state can be delicately controlled by external electric fields to modulate the conductance of channels. Ferroelectric devices are suitable for implementing neuromorphic devices as they can generate multi‐level current states by changing the polarization state using voltage pulses. Nevertheless, previously studied ferroelectric‐based materials, such as perovskite‐based ferroelectrics, have issues such as the disappearance of polarization with decreasing thickness, making them unsuitable for nonvolatile memory. Additionally, for lead zirconate titanate (PZT), a thickness of over 100 nm is required to obtain sufficient ferroelectricity, and contamination caused by the diffusion of volatile substances like Pb is not suitable for complementary metal‐oxide semiconductor (CMOS) processes, which is why it has not been commercialized despite being proposed prior to floating‐gate memory transistors due to its many drawbacks. The discovery of ferroelectricity in HfO_2_ compatible with CMOS in 2011 accelerated research on ferroelectric field‐effect transistors (FeFETs).^[^ ^9^ ^]^ Ferroelectric transistors based on HfO_2_ are very useful for fabricating devices with linear synaptic characteristics that are compatible with CMOS and have process scalability. Neuromorphic devices based on ferroelectrics have advantages in their changing characteristics, as the origin of conductivity modulation in FeFETs is controlled by partial polarization switching in the ferroelectric layer.^[^ ^10^ ^]^ Therefore, the controllability of channel conductance provides an opportunity to develop neuromorphic hardware based on ferroelectric analog synaptic transistors.

Due to their atomically thin bodies and excellent electronic tunability, 2D materials are being considered for application in neuromorphic computing systems, owing to their promising potential.^[^ ^11^ ^]^ Moreover, 2D semiconductors, with their thin, pristine bodies, offer atomically thin structures that are highly suitable for miniaturized processes. Additionally, 2D materials have demonstrated physical properties that are highly sensitive to external stimuli, making them promising candidates for mimicking synaptic functionality.^[^ ^12^ ^]^ SnS_2_ exhibits highly stable, non‐volatile switching behavior in response to electric pulses applied to the gate electrode, making it highly suitable as a non‐volatile memory channel.^[^ ^13^ ^]^ Recent studies have shown that SnS_2_ not only possesses excellent electrical characteristics but also exhibits a very low off‐current state, indicating its potential as a low‐power and high‐performance device.^[^ ^14^ ^]^

In this study, FeFETs were fabricated using 2D SnS_2_ film and nanoscale ferroelectric material to explore the potential of SnS_2_ for synaptic devices. The fabricated FeFET demonstrated multi‐level data storage capability and ferroelectric hysteresis with stable data retention. By showcasing analog potentiation and depression characteristics through the FeFET, we demonstrate the potential of utilizing nanoscale ferroelectric and semiconductor materials as synaptic devices for neuromorphic hardware systems. The fabricated FeFET device exhibited a stable and prolonged retention time, as well as a stable cycle characteristic, with a large window of 2 V during ±3 V voltage sweep at ambient conditions. We also show the potential of ferroelectric devices for artificial synapses by mimicking the dynamics of biological synapses such as excitatory/inhibitory postsynaptic currents (EPSC/IPSC), paired‐pulse facilitation (PPF), and long‐term potentiation/depression. Furthermore, Modified National Institute of Standards and Technology (MNIST) simulations were conducted to assess the applicability of hardware neural networks, and energy calculations were performed to evaluate the low‐power characteristics of our device.

## 2 Results

### 2.1 Characterization of the Fabricated Ferroelectric HfZrO_2_ (HZO) Film

**Figure 1:**
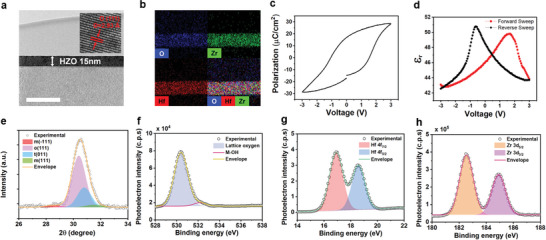
Characteristics of the deposited ferroelectric HZO film. a) HR‐TEM image showing the thickness (15 nm) of the ferroelectric HZO film and cross‐sectional image of orthorhombic HZO crystallite using HR‐TEM. Scale bar, 50 nm. The inset shows a magnified view of the atomic arrangement of orthorhombic HZO [111]. b) EDS mapping of the HZO cross‐section, depicting the distribution of the deposited elements (hafnium, zirconium, and oxygen). c) P‐V loops of HZO film. d) Permittivity – V loops of HZO film. e) Deconvoluted GIXRD pattern of the ferroelectric HZO film. High‐resolution XPS spectra of f) O 1s, g) Hf 4f, h) Zr 3d.

**Figure 2:**
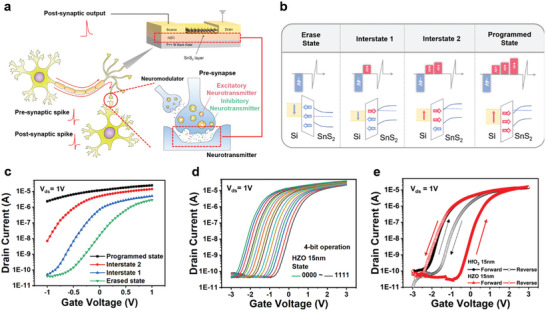
Synaptic Mimicry Ferroelectric HZO‐SnS_2_ Transistor. a) Schematic of the synaptic mimicry FeFET. b) Band diagram of the FeFET and a description of the modulation mechanism using pulse control. c) *I*_ds_‐*V*_g_ curve demonstrating conductance changes corresponding to band modulation. d) Operation of the 4‐bit SnS_2_ FeFET under refined PRG/ERS pulse control. e) Comparison of the *I*_ds_‐*V*_g_ loop of a ferroelectric HZO‐based SnS_2_ transistor, showing counterclockwise hysteresis characteristics, and a dielectric HfO_2_‐based SnS_2_ transistor, displaying clockwise hysteresis characteristics.

FeFETs employing an metal‐ferroelectric‐semiconductor (MFS) structure were successfully fabricated. The MFS structure was fabricated by depositing HZO on a heavily doped p‐type silicon substrate, with W electrodes deposited to characterize the ferroelectric properties. The memory features due to the polarization of HZO diminish beyond 30 nm thickness, making it indispensable to select an appropriate thickness of HZO.^[^ ^15^ ^]^ In order to implement a successful neuromorphic device, a targeted deposition thickness is 15 nm. **Figure** **1**a presents a transmission electron microscopy (TEM) image of the 15 nm thick HZO. Energy‐dispersive X‐ray spectroscopy (EDS) mapping confirmed a uniform distribution of each element (Hf, Zr, O) (Figure 1b). The fabricated HZO thin film processed an annealing process to induce ferroelectricity, and a sweep within the 3 V range confirmed a remnant polarization surpassing a 2P_r_ value of 30 µC cm^−2^ (Figure 1c). Capacitance‐voltage (C‐V) measurements in the 1 MHz frequency displayed satisfactory insulating characteristics along with a butterfly‐shaped result, which is shown in the ferroelectric layer (Figure 1d). The ferroelectricity of HZO, induced through the o‐phase, is demonstrated by grazing incidence X‐ray diffraction (GIXRD) analysis, showing all phases, including the o‐phase at 30.4 degrees and the t‐phase at 30.8 degrees (**Figure** **2**e). With the o‐phase accounting for 75%, we confirmed that it was well induced in a state where the m‐phase was suppressed. The surface chemical composition of the ferroelectric HZO grown by atomic layer deposition (ALD) was analyzed using X‐ray photoelectron Spectroscopy (XPS), and the spectrum was calibrated using the carbon 1s (C 1s) peak (284.6 eV) (Figure 1f–h). The spectral information for C 1s is provided in Figure S1 (Supporting Information). The high‐resolution O 1s spectrum exhibited a 5% oxygen vacancy layer, suppressing sufficient electron trap layers and successfully forming an oxide film capable of faithfully performing as a memory control layer. The established HZO film, in addition to EDS, had a 1:1 ratio, enabling the acquisition of high‐resolution spectra for Hf 4f and Zr 3d.

### 2.2 The Hysteresis Window of the SnS_2_/HZO Synaptic Transistor

The schematic representation of the fabricated SnS_2_/HZO FeFET is depicted in Figure 2a. SnS_2_ serves as the channel layer, while HZO acts as the ferroelectric gate layer for memory function. Figure 2b explains the mechanism contributing to the channel conductance variations as per the pulses between SnS_2_ and HZO. Initially, a negative voltage is used to create the erase state, which induces downward polarization, thus lowering the Fermi level of SnS_2_ and pushing the accumulated electrons in the channel to induce a high threshold voltage (*V*_th_). Subsequent input of positive voltage induces upward polarization, accumulating electrons in the channel, shifting the *V*_th_ in the negative direction, and thereby acquiring higher conductance. The outcome is shown as a 4‐state operation, and the actual experiment displays a 2‐bit step operation in Figure 2c. Through modulating factors like pulse width and voltage, it is feasible to achieve multi‐level data storage. In this experiment, we achieved the fabrication of a multi‐level FeFET functioning with a 4‐bit operation by achieving memory modulation with clearly distinguishable *V*_th_ (figure 2d). The memory window created through the multi‐level conductance states was able to reach more than 2.5 V. In Figure 2e, the observed counter‐clockwise hysteresis *I*_ds_‐*V*_g_ curve in the ±3 V sweep due to the ferroelectric property of HZO is contrary to the clockwise induction due to charge trapping phenomena by a SnS_2_ FET of the same thickness of HfO_2_. For multi‐level data, we successfully observed the holding characteristics of level states for a duration of 1 × 10^5^ s without significant degradation (Figure S2, Supporting Information). The fabricated HZO can have sufficiently remnant polarization characteristics, thus demonstrating its capacity to maintain data for over a decade reliably. It also showed residual polarization, indicating data storage characteristics without significant performance degradation over 10^7^ cycles, addressing durability issues. Furthermore, since SnS_2_ exhibits adequate response to HZO polarization, we were able to fabricate a more reliable synaptic transistor. With delicate pulse size control, we anticipate the possibility of driving a multi‐level transistor with higher bits operation.

### 2.3 The Synaptic Characteristics of the SnS_2_/HZO Transistor

**Figure 3:**
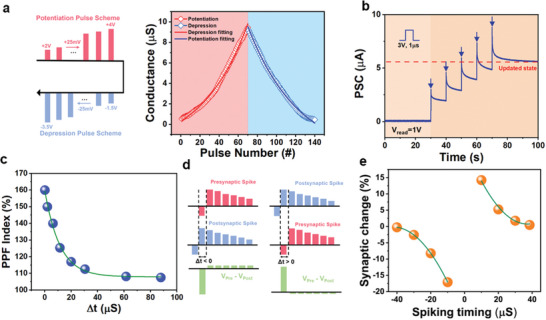
Synaptic Characteristics of SnS_2_ FeFET. a) Schematic of the waveform for long‐term potentiation and depression pulse trains and the linear potentiation/depression characteristics. b) Time dependence of PSC under the application of five pulses (amplitude: 3 V, width: 1 µs, period: 5 s). c) Dependence of PPF current index on pulse width. d) Waveform of pre‐ and post‐synaptic spikes with latency (Δ*t*) and voltage drop (*V*_pre_‐*V*_post_). e) STDP of the FeFET, exhibiting synaptic weight changes plotted against the relative timing of pre‐ and post‐synaptic spikes.

**Figure** **3**a demonstrates the synaptic characteristics acquired from long‐term potentiation and depression. By applying a pulse of 2–4 V amplitude to 70 features according to the pulses directed to the presynaptic neurons, we induced a continuous increase in conductance. Following the demonstration of the long‐term potentiation features, lower conductance was induced depending on the number of pulses ranging from −1.5 to −3.5 V for the long‐term depression features. Each feature was carried out with a fixed pulse width of 1 µs and a pulse interval of 10 µs, and the channel conductance in an environment of *V*_ds_ = 1 V was confirmed. The FeFET we fabricated allowed us to confirm 140 levels of multiple features, and excellent linear characteristics (*A*_p_ = −0.7239, *A*_d_ = −0.6026) were observed. We obtained a high conductance ratio of *G*_max_/*G*_min_ >100. To calculate the linearity of the increase/inhibition curve, we extracted the change in conductance using the applied pulse voltage and applied it to the following equation.^[^ ^10^, ^16^ ^]^

(1) $$G_{p}=B\left(1-e^{-P/A_{P}}\right)+G_{min}$$

(2) $$G_{d}=-B\left(1-e^{\frac{\left(P-P_{max}\right)}{A_{d}}}\right)+G_{max}$$

(3) $$B=\frac{\left(G_{max}-G_{min}\right)}{\left(1-e^{-P_{max}/A_{p,d}}\right)}$$

In this regard, *G*_p_, *G*_d_, *P*, *P*_max_, and *A* represent ascending conductance, descending conductance, the number of pulses, the maximum number of pulses, and ascending/descending linearity, respectively. High linearity, *G*_max_/*G*_min_, and the number of multi‐levels is the most critical factor determining the recognition rate. Neurons and synapses are the smallest units of learning and memory in the brain, and they are also the most intensively researched in neuromorphic computing systems.^[^ ^17^ ^]^ In biological systems, signal transmission efficiency from one neuron to another is determined by synaptic weights, which can be well‐regulated by deliberate stimuli or training. Changes in synaptic weight between presynaptic neurons and postsynaptic neurons are known as synaptic plasticity, including short‐term plasticity (STP), long‐term plasticity (LTP), and spike‐timing‐dependent plasticity (STDP).^[^ ^18^ ^]^ SnS_2_ FeFET showed that the postsynaptic current (PSC) obtained by two pre‐spikes (4 V, 1 µs, Δ *t* = 2 µs) were 4.1 and 6.6 µA, respectively, and the PPF index (*A*_2_/*A*_1_) was 162% (Figure S3, Supporting Information). The temporarily increased PSC decreased after the stimulus ended, but the PSC was higher than before the spike was applied, and a higher PSC was induced after continuous stimuli (Figure 3b). This can be attributed to the polarization transition induced by the spike in randomly arranged ferroelectric domains, and the up‐state polarization increases the conductivity of the channel. After the stimulus, some polarizations can be explained by the phenomenon of decreasing conductivity as it is rearranged randomly. Due to the residual polarization induced by external spikes, continuous stimuli can gradually increase the PSC. When the energy required to change the ferroelectric domain is minimized, a synaptic‐like event is activated, consuming 48 aJ (Figure S4, Supporting Information). This facilitates control with low‐power energy consumption, allowing for operation at levels as low as 10 fJ, even at peak performance, which is more efficient than human synapses. This is a comprehensive result of the low‐power operation of 2D channels and the rapid switching of ferroelectrics within 1 µs. Additionally, the switching operation speed is 10^4^ times faster than the 10 ms speed of human synapses. PPF is a phenomenon in the nervous system where the PSC induced by the presynaptic spike increases when the presynaptic spike closely follows the previous spike, which is very important for decoding temporary information in the nervous system.^[^ ^19^ ^]^ PPF characteristics were examined within a range of 1–90 µs pulse intervals. A presynaptic spike (+4 V, 1 µs) was applied to the presynaptic neuron, and the read voltage (*V*_ds_ = 1 V) was applied following two consecutive presynaptic spikes. As the pulse interval increases, the polarization relaxation time also increases, reducing the excitation state of the synapse significantly (Figure 3c). Conversely, as the pulse interval decreases, the relaxation time of the polarization switched by the first spike decreases, causing more domains to be switched by the subsequent spike. The PSC value, and in turn, the PPF index, increases as the A_2_ pulse interval decreases. These PPF characteristics demonstrate the ability to mimic biological synapses by implementing STP.^[^ ^20^ ^]^ Following the demonstration of the ability to implement STP, LTP, and STDP characteristics were explored. In the nervous system, synaptic connections are regulated by the causality between neurons, contributing to the brain's computation and memory formation.^[^ ^21^ ^]^ Among various learning rules, STDP stands out as it modifies synaptic weights based on the time difference between presynaptic and postsynaptic spikes, as well as voltage differences (*V*_pre_ – *V*_post_). If the presynaptic neuron operates before the postsynaptic neuron, the synaptic weight goes up. Conversely, if the presynaptic neuron fires after the postsynaptic neuron, the weight decreases. The increase in weight is attributed to long‐term potentiation, while the decrease is linked to long‐term depression. The effectiveness of the STDP learning rule was assessed by observing its implementation in FeFET, as depicted in Figure 3d. The overlapping waveforms from both presynaptic and postsynaptic spikes lead to polarization changes based on their timing difference. The change in conductance was observed by applying a read voltage to the postsynaptic neuron before and after the voltage shift. The partial polarization of the HZO layer shift, which increases device conductance as Δ*t* reduces due to more domain transitions, is suitable for emulating the synaptic strength changes based on time gaps, as seen in Figure 3e.

**Figure 4:**
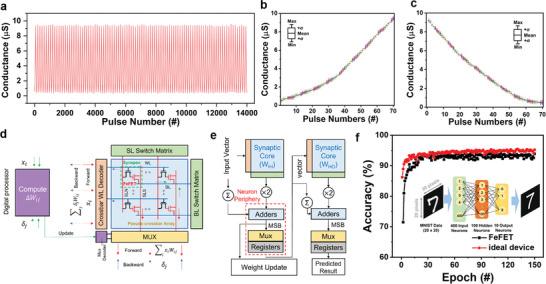
Potential for Synaptic Mimicry Devices Based on Ferroelectric HZO‐SnS_2_ Transistors. a) Stability test for the long‐term potentiation/depression curve applied to the presynaptic neuron over 100 cycles. b) Distribution of long‐term potentiation and c) long‐term depression over 100 cycles. d) Schematic overview of in‐memory computing simulation. e) MLP network diagram for simulation operation. f) Comparison of the accuracy of the MNIST handwritten image classification between the ideal device and the SnS_2_ FeFET device.

To ensure the reliability and reproducibility of the experiment, 100 cycle tests (14,000 pulses) were conducted and confirmed that the linearity of the device and the conductance of the channel varied consistently without significant deviation (**Figure** **4**a–c). Additionally, the examination of device‐to‐device variation among the fabricated FeFET devices demonstrated the reliability of FeFET devices (Figure S5, Supporting Information). These results allowed us to perform a simulation of artificial neural networks (ANN) based on multi‐layer perceptron (MLP) via FeFET array using Neurosims 3.0.^[^ ^22^ ^]^ Before being mapped onto the synaptic array, the weights of the deep learning algorithm need to be quantized, and the conventional hardware implementation of n‐bit quantized weights groups n‐binary synaptic cells to represent the n‐bit weight value. The weights are then modified through a learning process involving a weight update process (Figure 4d,e). The calculated values from the synaptic core are ultimately used to perform the ANN, here deriving prediction values for MNIST data. The MNIST data of 20 × 20 size grayscale handwritten digit patterns were used for both training (60,000 sets) and testing (10,000 sets). Based on the long‐term potentiation/depression curves, our devices can achieve a maximum accuracy of 94%, which is remarkably close to the 95% obtained from an ideal device (Figure 4f).

## 3 Conclusion

In this research, 2D analog synaptic FETs, using ferroelectric HZO and 2D semiconductor (SnS_2_), were fabricated and characterized. Our device demonstrates in‐memory computing with multi‐level data storage attributes (>7‐bit) and successfully mimics biological characteristics such as EPSC/IPSC, PPF, and STDP. SnS_2_ FeFET exhibited excellent stability, excellent linearity, and high *G*_max_/*G*_min_ (>10^5^). High‐efficiency and ultralow‐power neuromorphic hardware, which is in sub‐femtojoule (48 aJ/spike) and fast response (1 µs), which is 104 folds faster than human synapse (≈10 ms) due to gradually switching ferroelectric domains. Furthermore, simulations of the artificial neural networks in our device demonstrate a 94% pattern recognition rate on the MNIST handwritten dataset, leveraging its in‐memory computing attributes. The integrated structure of memory and computing in the FeFET enables a highly compact and energy‐efficient device. **Table** **1** shows the characteristics of our device alongside the typical features of recently reported three‐terminal synaptic transistors. As compared in Table 1, the overall superior results demonstrated in this study indicate that the SnS_2_ and HZO structures can be attractive candidates for synaptic architectures in neuromorphic computing. Moreover, the device designed incorporates 2D materials and ferroelectric HZO with high permittivity, allowing for an ultra‐thin body, and thus holds great promise for future advancements.

**Table 1:** Performance comparison of various synaptic transistors for neuromorphic computing.

Materials Structure	On/off	Speed	State	Endurance [pulse]	Power consumption	MNIST Accuracy	Symmetry	Reference
InSe/InO_x_/SiO_2_	>10^5^	50 ms	200	100	12 pJ	70%	asymmetry	[23]
MoTe_2_/HZO	>10^3^	1 s	100	100	N/A	80%	symmetry	[24]
MoO_x_/MoS_2_/h‐BN	>10^5^	100 ns	60	1,440	0.1fJ	91%	symmetry	[25]
IZTO/HZO	>10^6^	10 µs	128	10^4^	N/A	91%	symmetry	[26]
IGZO/HfO_2_/HZO	>10^6^	0.5 ms	200	2×10^4^	4.35 pJ	94%	Asymmetry	[27]
Te/h‐BN/Gr	> 10	10 µs	120	N/A	N/A	90%	Asymmetry	[28]
SnS_2_/HZO	> 10^5^	1 µs	140	1.4×10^4^	48 aJ	94%	symmetry	This work

## 4 Experimental Section

### Fabrication of Ferroelectric SnS_2_ FET

In this study, the proposed device was designed to possess an MFS structure consisting of SnS_2_ /HZO/P^++^ Si. After cleaning with BOE, rinsing with deionized water was carried out on the P^++^ Si substrate. A layer of HZO was then deposited at a temperature of 270 °C using ALD. Tetrakis(dimethylamido)hafnium (TEMAH), tetrakis(dimethylamino)zirconium (TDMAZ), and ozone were used as the hafnium precursor, zirconium precursor, and oxygen source, respectively. The ALD cycle ratio of HfO_2_ and ZrO_2_ was set to 1:1, resulting in the deposition of a thin film with a thickness of ≈15 nm. A rapid thermal annealing (RTA) process was then conducted for 30 s at 550 °C in a nitrogen gas environment to induce ferroelectricity. Subsequently, a SnS_2_ flake was transferred through mechanical exfoliation, followed by the deposition of Ti/Au (10 nm/50 nm) through electron beam evaporation. Finally, the sample was annealed for 1 h at 100 °C to desorb water and oxygen molecules present on the surface.

### Characterization

All characteristics of the FeFET were measured under ambient conditions. Electrical characteristics were measured using a semiconductor parameter analyzer (4200‐scs, Keithley). The thickness of HZO was measured using TEM, and its composition was analyzed through EDS. The thickness of SnS_2_ was measured at a scan rate of 0.3 Hz using atomic force microscopy (AFM, NX‐10, Park systems). The polarization‐voltage and capacitance‐voltage curves were measured using a pulse measurement device (B1500‐A, Keysight). The ferroelectric characteristics of the deposited HZO were obtained from P‐V hysteresis curves measured by a Ferroelectric tester (Premier ll, Radiant Technologies, Inc).

All simulations were performed using the open‐source software NeuroSims 3.0 through C++ code in a Linux system equipped with GCC and GNU C libraries. The simulated MLP neural network was constructed with 400 input neurons, 100 hidden neurons, and 10 output neurons. The 400 input neurons received character data corresponding to 20 × 20 MNIST images, and the 10 output neurons corresponded to 10 number classes from 0 to 9. In this simulation, *G*_max_/*G*_min_, linearity, and inter/intra‐device variability of the FeFET were considered as synaptic device characteristics. The simulation of the ideal synaptic network utilized ideal synaptic characteristics, including *G*_max_/*G*_min_ = 50 and perfectly linear conductance modulation with 65 conductance states.

## Conflict of Interest

The authors declare no conflict of interest.

## Supporting information

Supporting Information

## Data Availability

The data that support the findings of this study are available from the corresponding author upon reasonable request.
