# Supplementary material for: Ferroelectric 2D SnS2 Analog Synaptic FET
Source: Adv Sci (Weinh). 2024 Feb 20;11(16):2308588. doi: 10.1002/advs.202308588 (PMC11040367; doi:10.1002/advs.202308588)
Supplement: Supplementary file 1 — Supporting Information [file ADVS-11-2308588-s001.pdf]

ADVANCED SCIENCE
Open Access

Supporting Information

for *Adv. Sci.*, DOI 10.1002/advs.202308588

Ferroelectric 2D SnS_2 Analog Synaptic FET

*Chong-Myeong Song, Dongha Kim, Shinbuhm Lee and Hyuk-Jun Kwon**

Supporting Information

Ferroelectric 2D SnS_2 analog synaptic FET

*Chong-Myeong Song, Dongha Kim, Shinbuhm Lee, and Hyuk-Jun Kwon**

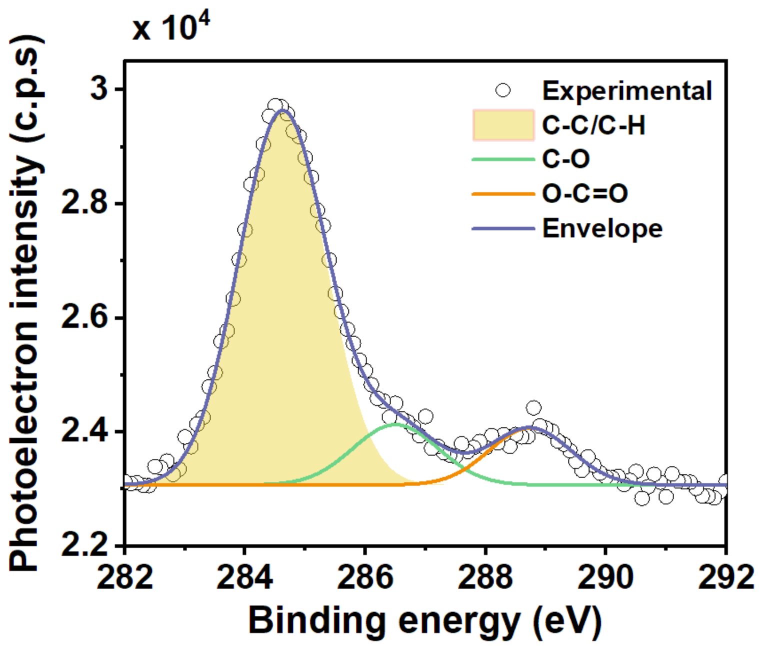

Figure S1. High-resolution XPS spectra of C 1s of HZO film.

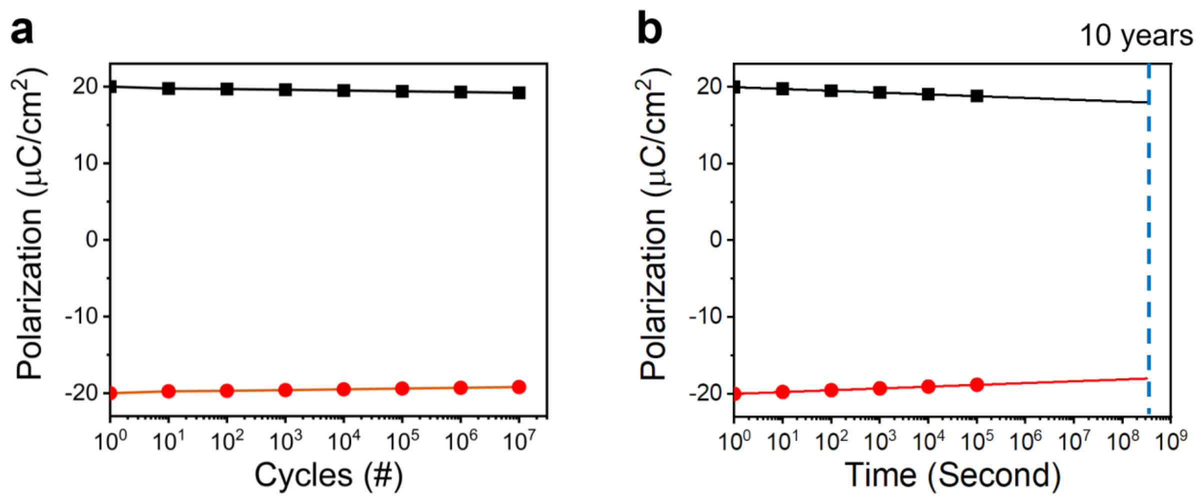

Figure S2. a) Endurance of HZO capacitors using the electric field of 3 MV/cm. b) Polarization retention of HZO capacitors.

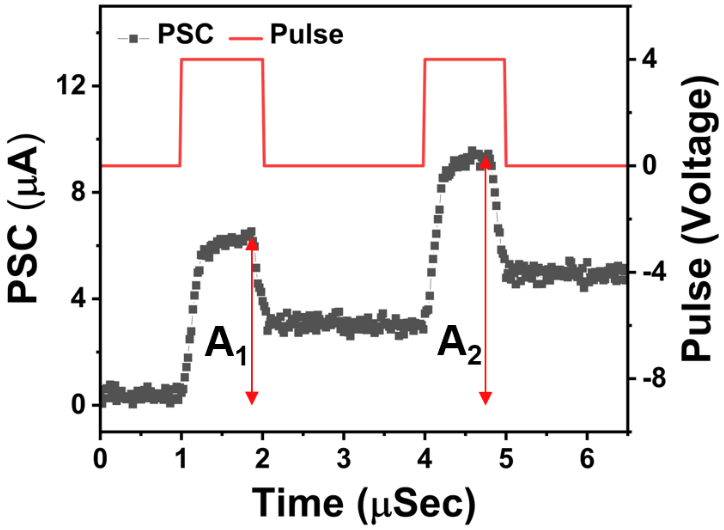

Figure S3. PPF behavior in SnS_2 FeFET.

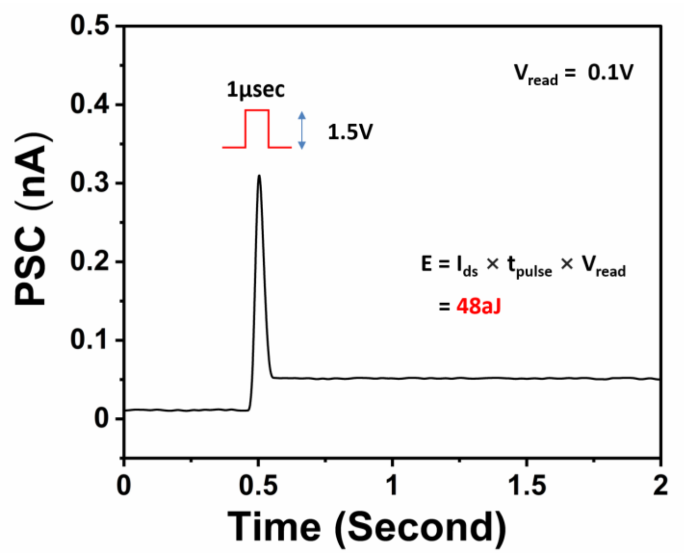

Figure S4. EPSC responses by pre-synaptic spike (1.5 V, 1 μs) and calculation of energy consumption per spike (Read at V_{read} = 0.1 V and V_{gs} = -0.5 V).

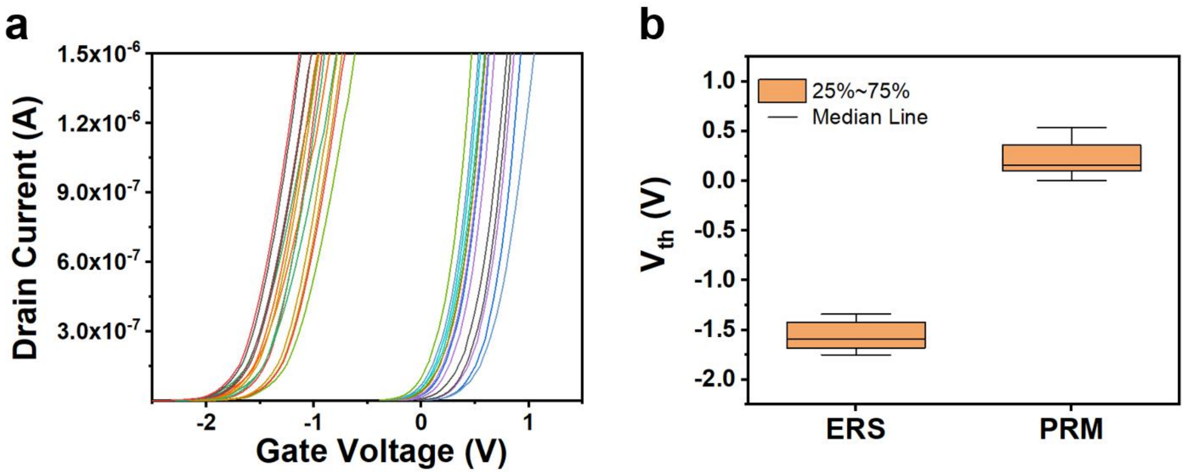

Figure S5. Device-to-device variations of the performance of HZO-SnS_2 FeFETs. The drain current is normalized by L/W.

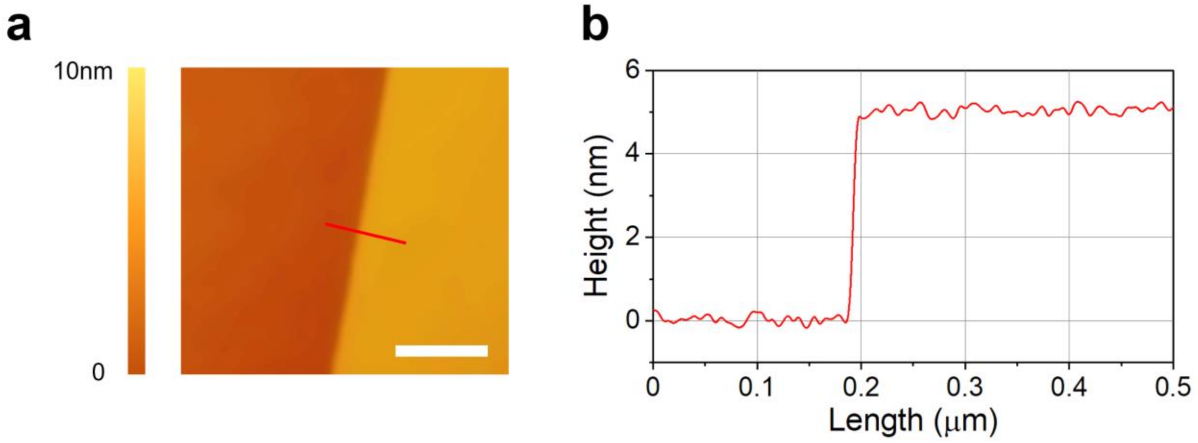

Figure S6. a) AFM (Atomic Force Microscopy) image of the SnS_2 flake. Scale bar, 0.5 μm. b) Cross-sectional plot along the red line.
